# COVID-19 pneumonia: the great radiological mimicker

**DOI:** 10.1186/s13244-020-00933-z

**Published:** 2020-11-23

**Authors:** Selin Ardali Duzgun, Gamze Durhan, Figen Basaran Demirkazik, Meltem Gulsun Akpinar, Orhan Macit Ariyurek

**Affiliations:** Department of Radiology, School of Medicine, Tıp Fakültesi Hastanesi, Hacettepe University, 06100 Sıhhiye, Ankara, Turkey

**Keywords:** COVID-19, Pneumonia, Mimicker, Chest CT, Differential diagnoses

## Abstract

Coronavirus disease 2019 (COVID-19), caused by severe acute respiratory syndrome coronavirus 2 (SARS-CoV-2), has rapidly spread worldwide since December 2019. Although the reference diagnostic test is a real-time reverse transcription-polymerase chain reaction (RT-PCR), chest-computed tomography (CT) has been frequently used in diagnosis because of the low sensitivity rates of RT-PCR. CT findings of COVID-19 are well described in the literature and include predominantly peripheral, bilateral ground-glass opacities (GGOs), combination of GGOs with consolidations, and/or septal thickening creating a “crazy-paving” pattern. Longitudinal changes of typical CT findings and less reported findings (air bronchograms, CT halo sign, and reverse halo sign) may mimic a wide range of lung pathologies radiologically. Moreover, accompanying and underlying lung abnormalities may interfere with the CT findings of COVID-19 pneumonia. The diseases that COVID-19 pneumonia may mimic can be broadly classified as infectious or non-infectious diseases (pulmonary edema, hemorrhage, neoplasms, organizing pneumonia, pulmonary alveolar proteinosis, sarcoidosis, pulmonary infarction, interstitial lung diseases, and aspiration pneumonia). We summarize the imaging findings of COVID-19 and the aforementioned lung pathologies that COVID-19 pneumonia may mimic. We also discuss the features that may aid in the differential diagnosis, as the disease continues to spread and will be one of our main differential diagnoses some time more.

## Introduction

Severe acute respiratory syndrome coronavirus 2 (SARS-CoV-2), a novel coronavirus, was found to be associated with a pneumonia outbreak firstly reported in Wuhan, China, in December 2019 [1, 2]. The disease subsequently called Coronavirus Disease 2019 (COVID-19) has caused a lot of cases worldwide and was declared as a pandemic by the World Health Organization (WHO) on March 11, 2020 [3].

The reference diagnostic test for COVID-19 pneumonia is real-time reverse transcription-polymerase chain reaction (RT-PCR). The specificity of RT-PCR is approximately 95%, but the sensitivity of RT-PCR at the initial presentation is 60–71% because of kit performance, sampling and transportation limitations [4–6]. Because of these low sensitivity rates and the need for rapid diagnosis, non-contrast chest-computed tomography (CT) has been frequently used in the current pandemic condition. Also, several cases with initial negative RT-PCR results are reported to have positive chest CT findings [4]. Although recent studies have reported the high sensitivity of chest CT for diagnosis with RT-PCR as a reference, the specificity of CT is relatively low. In a recent meta-analysis, chest CT had a pooled sensitivity of 94% and a specificity of 37% [4, 5, 7]. This may be explained by the fact that besides previously reported typical appearance, COVID-19 has various CT findings, and some chest CT findings of COVID-19 may closely resemble the imaging findings of some other pathologies presenting with airspace disease.

In this pictorial review, CT findings of COVID-19 pneumonia and imaging features of COVID-19 pneumonia that may mimic other infectious and non-infectious diseases will be summarized.

## CT findings of COVID-19 pneumonia

Typical CT findings of COVID-19 pneumonia are predominantly peripheral, bilateral ground-glass opacities (GGOs), consolidations, combination of GGOs with consolidations, and GGOs superimposed with interlobular/intralobular septal thickening creating a “crazy-paving” pattern and subpleural linear opacities. Air bronchograms, vascular enlargement, CT halo sign, and reverse halo sign are also reported. Cavitation, pleural or pericardial effusion, and lymphadenopathy are rarely observed [8–11].

**Fig. 1:**
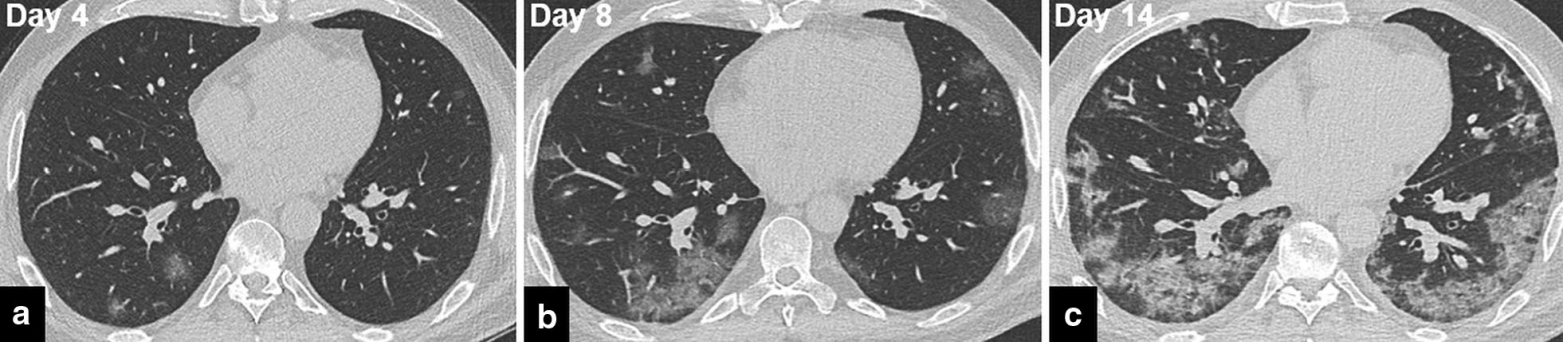
CT findings of COVID-19 pneumonia. **a** The first CT scan obtained on illness day 4 revealed patchy GGOs in both the lungs. **b** On day 8, the number and size of GGOs increased. **c** Consolidations were the dominant CT finding on day 14

Findings vary and usually progress during the course of the disease. In the early phase, the predominant finding is unilateral or bilateral small peripheral GGOs. While the size and number of GGOs and the number of affected lobes increase, crazy-paving pattern and consolidations appear as the disease progresses. Consolidations become denser at the peak stage (Fig. 1). Approximately after 2 weeks, opacities start to resolve gradually, and residual subpleural curvilinear lines, fibrous stripes, and GGOs may be seen [9, 12, 13].

## Infectious causes

Differentiation of COVID-19 pneumonia from other causes of infection is sometimes challenging, especially when it comes to viral pneumonia and other atypical pneumonia. Bai et al. reported some findings in favor of COVID-19 pneumonia rather than non-COVID pneumonia, such as the presence of GGOs, peripheral distribution, reverse halo sign, and vascular enlargement. Pleural effusion and thickening, lymphadenopathy, and central distribution are less likely to be seen in COVID-19 pneumonia [14]. By contrast, some typical findings described for COVID-19 pneumonia can be seen in non-COVID pneumonia or other pathologies. For example, both the reverse halo sign and CT halo sign have also been previously described in fungal infections and tuberculosis [15–17].

The diagnostic association of CT findings for different infectious diseases is summarized in Table 1.

**Table 1:** CT findings of COVID-19 pneumonia and other infectious diseases

CT finding	COVID-19 pneumonia	Non-COVID viral pneumonia	Bacterial pneumonia	PJP	Fungal pneumonia
GGO	+++	++	+	+++	+
Consolidation	++	++	+++	0	+
Centrilobular nodular opacities	−−−	++	++	−	+
Crazy-paving	++	++	+	++	−
Lesion distribution					
Peripheral	+++	++	+	+	0
Lower zone	+++	++	+	+	0
Rounded morphology	+++	+	+	−−	++
Cavitation	−−−	−	+	−	++
Pleural effusion	−−	+	++	0	+
Lymphadenopathy	−−	+	++	0	+

The most common findings of aforementioned disease groups are presented. Signs indicate the strength of relation between the CT finding and diagnosis; in a range of (+++) and (−−−); (+++) indicating the strongest association

### Viral pneumonia

**Fig. 2:**
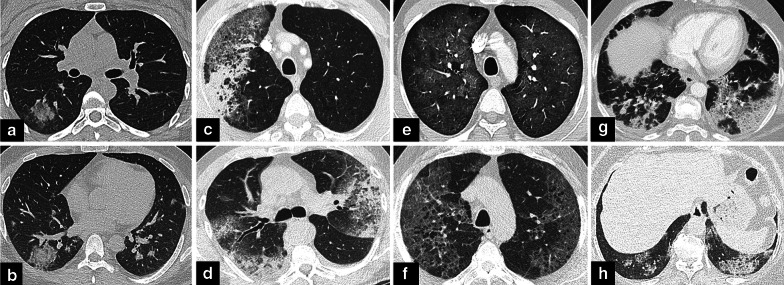
Other infectious diseases that COVID-19 pneumonia may mimic. **a** Axial CT image shows a consolidation with rounded morphology in the right lower lobe. Hemophilus influenza was detected in the respiratory panel. **b** Similar round consolidation is observed in the right upper lobe. The patient was tested positive on RT-PCR for COVID-19 pneumonia. The respiratory panel was negative for other causes. **c** Axial CT image shows GGOs with associated consolidations in a patient with Hemophilus influenza pneumonia. **d** In a patient diagnosed with COVID-19 pneumonia, bilateral GGOs with consolidations are observed on axial CT image similar to (**c**). **e** Axial CT image shows GGOs in bilateral upper lobes in an immunosuppressed patient compatible with PJP infection. **f** Bilateral upper lobe GGOs in a patient diagnosed with COVID-19 pneumonia. GGOs superimposed on emphysematous parenchyma may interfere with PJP. Subpleural sparing is in favor of PJP. **g** Axial CT image shows bilateral consolidative opacities in lower lobes in a patient diagnosed with influenza A pneumonia. **h** In a patient diagnosed with COVID-19 pneumonia, bilateral opacities are observed in lower lobes

Chest CT findings of a wide range of viruses have been previously described, such as influenza virus, parainfluenza virus, adenovirus, respiratory syncytial virus (RSV), cytomegalovirus (CMV), human metapneumovirus (HMPV), and other coronaviruses. Viral pneumonia commonly manifests as interstitial pneumonia with nonspecific imaging findings, such as GGOs, patchy consolidations, peribronchovascular thickening, centrilobular nodular opacities, a “tree-in-bud” pattern, and interlobular septal thickening [18, 19] (Fig. 2). Unlike in other viral pneumonia, centrilobular nodular opacities are not common in COVID-19 pneumonia.

Some typical findings of COVID-19 pneumonia, such as the subpleural distribution of opacities and a crazy-paving pattern, were also described for pneumonia caused by other coronaviruses, severe acute respiratory syndrome virus (SARS), and middle east respiratory syndrome virus (MERS), that caused outbreaks previously [20–22]. The absence of cavitation, pleural effusion, and lymphadenopathy are common in both COVID-19 pneumonia and SARS [20].

### Bacterial pneumonia

Although viral and bacterial pneumonia have overlapping imaging findings, the latter generally causes lobar/segmental pneumonia or bronchopneumonia and focal or multifocal consolidations. Less commonly, GGOs may be seen. Peribronchial thickening, centrilobular nodular opacities, and pleural effusion are much more frequent in bacterial pneumonia than in COVID-19 pneumonia [18, 23] (Fig. 2).

### Pneumocystis jirovecii pneumonia (PJP)

GGOs and crazy-paving pattern are also predominant in PJP, especially in an immunosuppressed host. Relative central lung involvement, upper lobe predilection, and pulmonary cysts in PJP, unlike in COVID-19 pneumonia, can help in differential diagnosis [24] (Fig. 2).

### Fungal pneumonia

**Fig. 3:**
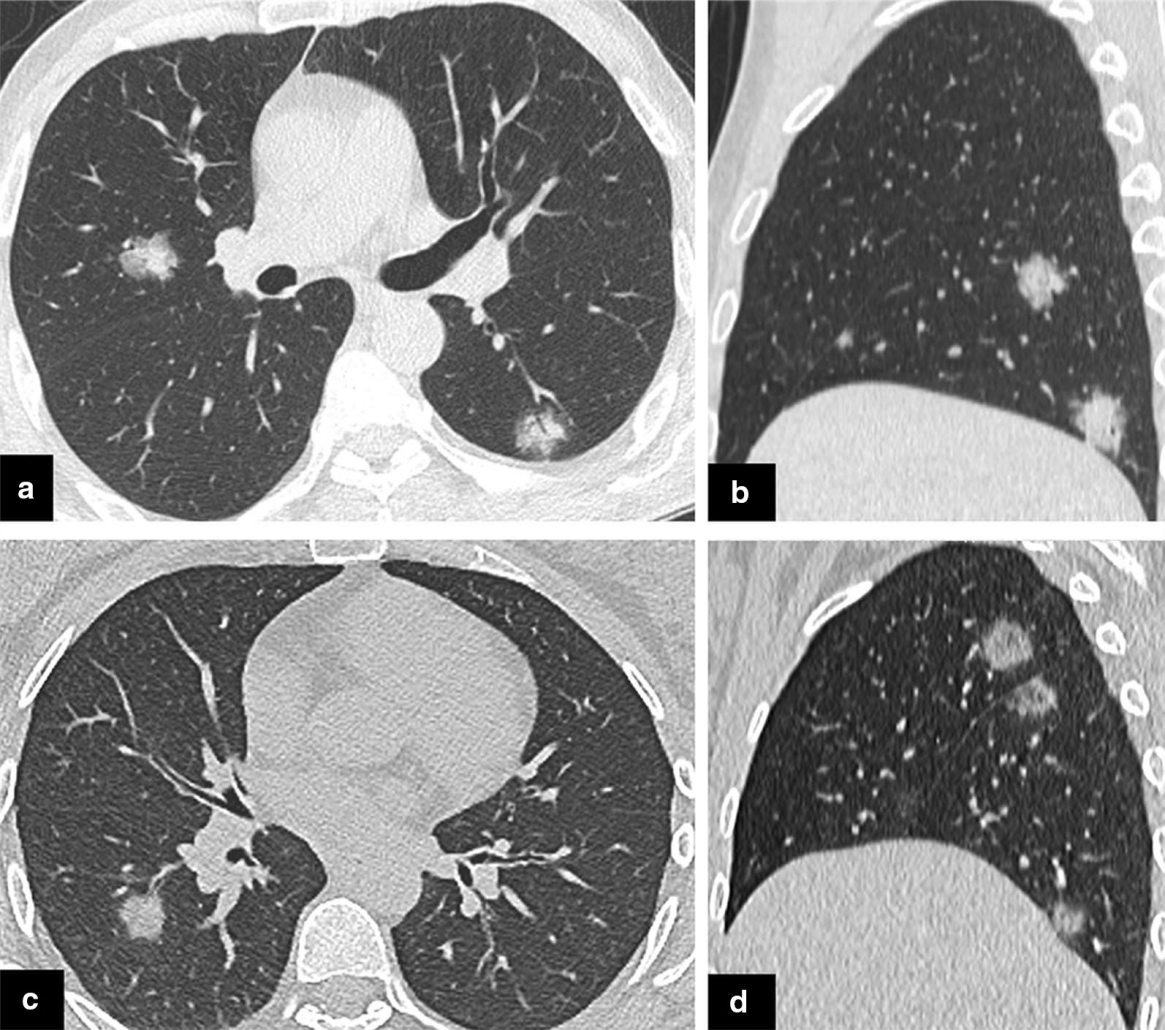
Fungal infection and COVID-19 pneumonia. **a**, **b** Axial (**a**) and sagittal (**b**) CT images show bilateral nodular opacities in a patient diagnosed with microbiologically proven aspergillosis. **c**, **d** Axial (**c**) and sagittal (**d**) CT images show nodular opacities in a COVID-19 patient. Note that the central part of the opacities in COVID-19 pneumonia has a relatively lower density (**d**)

Fungal pneumonia may have various imaging findings, such as GGOs, nodular opacities, a tree-in-bud pattern, and cavitating consolidations [25, 26]. A CT halo sign in which the surrounding GGOs represent hemorrhage has been commonly described in angioinvasive aspergillosis. In addition, a CT halo sign may be seen in candidiasis, cryptococcosis, and coccidioidomycosis [17, 26]. The reverse halo sign was previously reported in mucormycosis, invasive pulmonary aspergillosis, paracoccidioidomycosis, histoplasmosis, and cryptococcosis [15]. Although some CT findings of fungal pneumonia overlap with those of COVID-19 pneumonia, the presence of centrilobular nodular opacities, cavitation, chest wall invasion, pleural effusion, and lymphadenopathy favor the diagnosis of a fungal infection [25, 26] (Fig. 3).

### Super-infection/co-infection

**Fig. 4:**
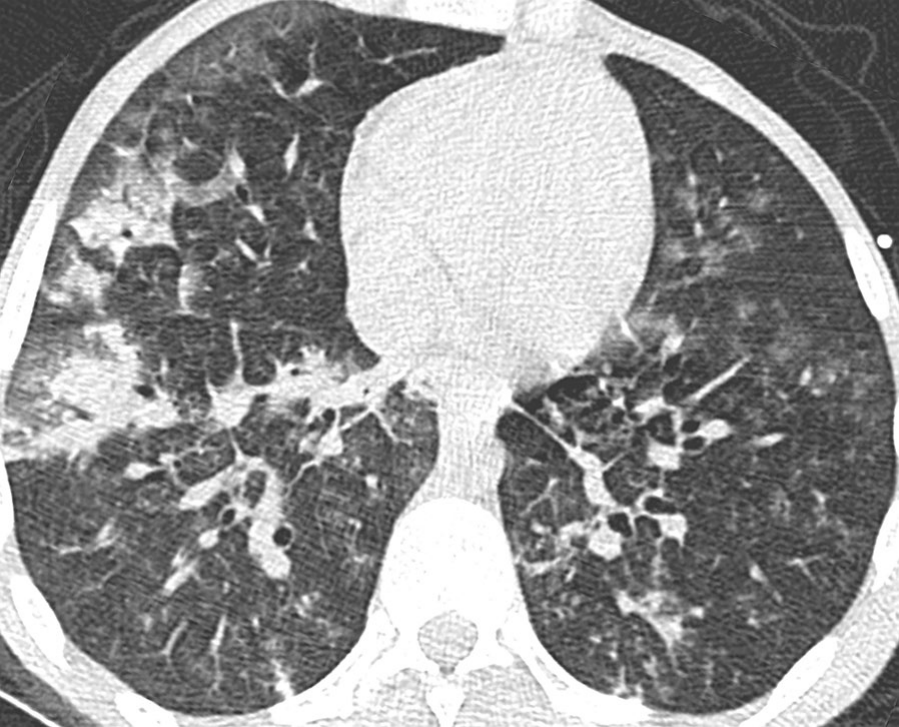
Bacterial super-infection in COVID-19 pneumonia. CT scan was obtained in a patient previously diagnosed with COVID-19 pneumonia upon clinical deterioration. Axial CT image shows bilateral peribronchovascular nodular GGOs, centrilobular nodular opacities, and consolidations in the right lung. Peripherally located GGOs are observed in the right middle lobe. Respiratory tract sample was positive for Hemophilus influenza

A recent meta-analysis reported bacterial and viral co-infection rates among hospitalized COVID-19 patients as 7% and 3%, respectively. Moreover, intensive care unit (ICU) patients showed a higher bacterial co-infection rate (14%) [27]. Although COVID-19 pneumonia may mimic other lung infections, the presence of atypical findings, such as pleural effusion, lymphadenopathy, lobar consolidation, or centrilobular nodular opacities, should raise a concern about super-infection or co-infection in patients diagnosed with COVID-19 pneumonia [18] (Fig. 4).

## Non-infectious causes

The diagnostic association of CT findings for COVID-19 pneumonia and different non-infectious differential diagnoses are summarized in Table 2.

**Table 2:** CT findings of COVID-19 pneumonia and non-infectious diseases

CT finding	COVID-19 pneumonia	Pulmonary Edema	Pulmonary Hemorrhage	Neoplasms	Organizing pneumonia	PAP	Sarcoidosis	Infarction	Interstitial diseases	Aspiration pneumonia
GGO	+++	+++	+++	+	++	+++	+	+	++	+
Consolidation	++	++	++	++	++	++	+	++	+	++
Centrilobular nodular opacities	−−−	−	+	+	0	−−	++	−−−	+	++
Crazy-paving	++	+	++	+	+	+++	+	−−−	+	0
Lesion distribution										
Peripheral	+++	−−	+	+	++	−	0	+++	++	++
Lower zone	+++	+	+	+	++	0	−	++	++	+++
Rounded morphology	+++	−	++	++	+	−	+	+	0	+
Cavitation	−−−	−−−	0	+	−	−	−	+	−−	++
Pleural effusion	−−	++	+	++	−	−−	−	++	−	+
Lymphadenopathy	−−	+	0	++	0	−	+++	0	+	+

The most common findings of aforementioned disease groups are presented. Signs indicate the strength of relation between the CT finding and diagnosis; in a range of (+++) and (−−−); (+++) indicating the strongest association.

### Pulmonary edema

**Fig. 5:**
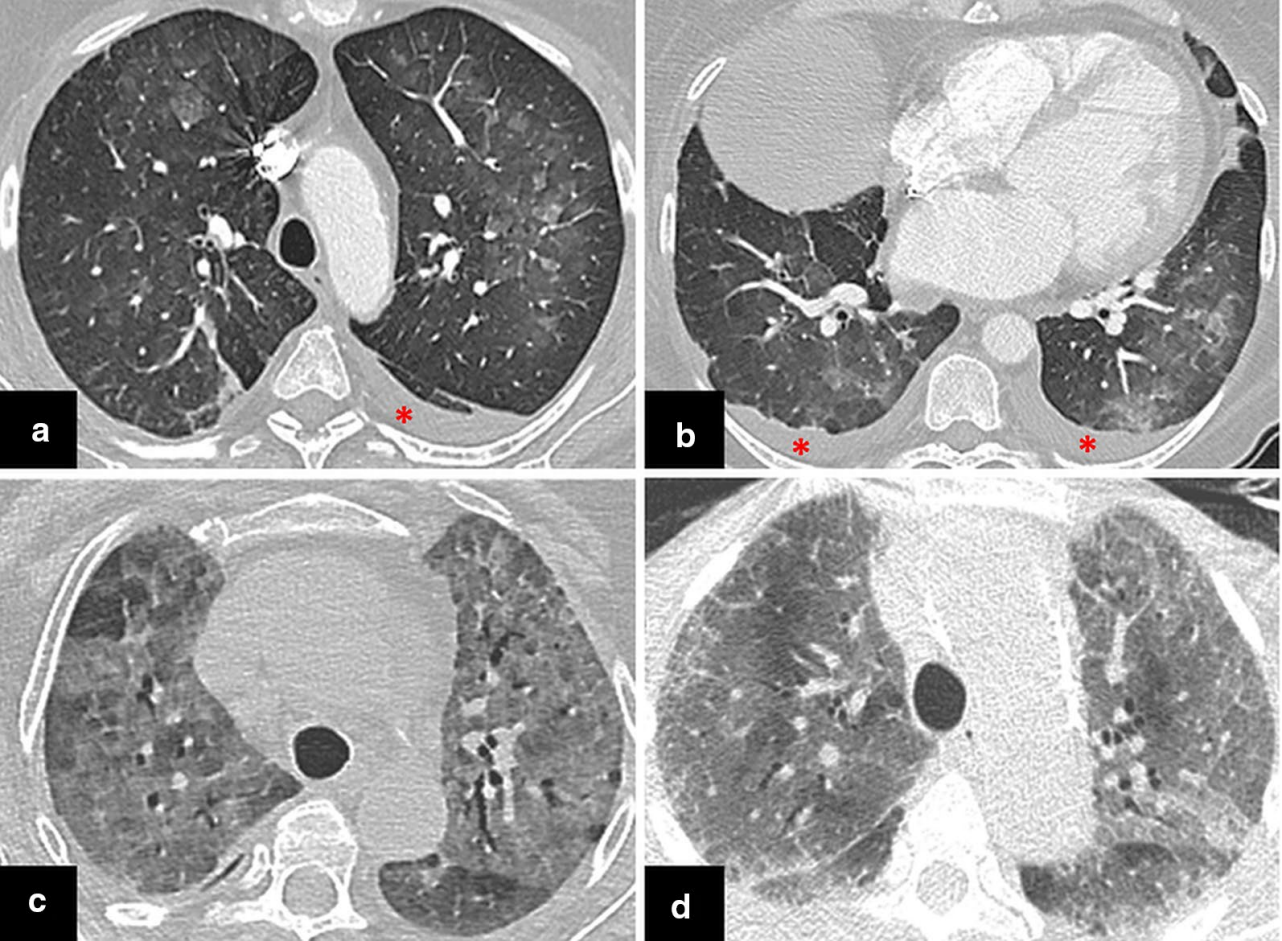
Distribution of ground-glass opacities in pulmonary edema and COVID-19 pneumonia. **a**, **b** Bilateral predominantly central GGOs are observed in a patient with pulmonary edema. Subpleural sparing is seen. There is also cardiomegaly and bilateral pleural effusion (asterisks). **c**, **d** In two different COVID-19 patients, diffuse GGOs are seen in bilateral upper lobes on axial CT images. The absence of ancillary findings such as pleural effusion and cardiomegaly may help in the differential diagnosis

In pulmonary edema, diffuse or patchy GGOs can be observed similar to those in COVID-19 pneumonia. However, the central and gravitational predominance of GGOs and the other accompanying findings, such as interlobular septal thickening, vascular redistribution, peribronchovascular cuffing, cardiomegaly, and pleural effusion, can help in distinguishing cardiogenic pulmonary edema from COVID-19 pneumonia [28] (Fig. 5).

By contrast, COVID-19-related GGOs may interfere with asymmetrical edema findings such as mitral regurgitation-associated edema or neurogenic edema. In mitral regurgitation due to right upper pulmonary vein-directed reflux, edema findings tend to occur in the right upper lobe [29]. Neurogenic pulmonary edema can be seen in patients with brain injury, and in nearly half of the cases, parenchymal opacities are located in apices [30].

### Pulmonary hemorrhage

**Fig. 6:**
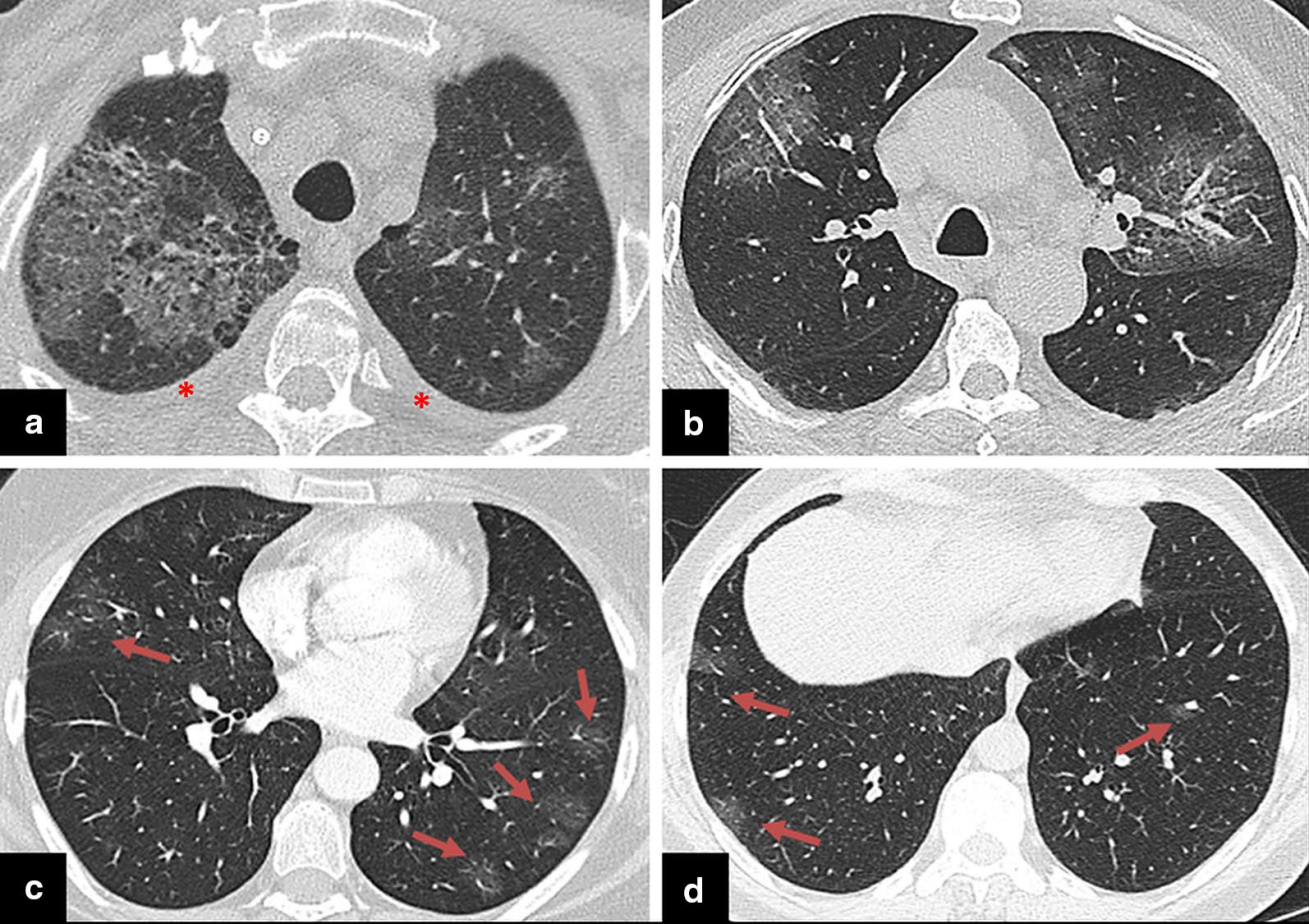
COVID-19 pneumonia-mimicking vasculitic diseases. **a** Axial CT image of a patient diagnosed with granulomatosis with polyangiitis shows bilateral upper lobe GGOs with superimposed septal thickenings due to hemorrhage. Bilateral minimal pleural effusion is seen (asterisks). **b** Similar bilateral upper lobe GGOs with superimposed septal thickenings are observed in COVID-19 pneumonia. **c** Bilateral mostly peripheral subtle GGOs in a patient diagnosed with eosinophilic granulomatosis with polyangiitis (Churg-Strauss)(arrows). **d** Peripheral subtle GGOs are seen in a COVID-19 patient as an early finding (arrows)

GGOs and consolidative opacities are also commonly seen in many causes of alveolar hemorrhage, such as collagen-vascular diseases, idiopathic pulmonary hemosiderosis, vasculitis (eosinophilic granulomatosis with polyangiitis, Goodpasture syndrome, granulomatosis with polyangiitis), pulmonary contusion, and anticoagulation therapy [31, 32]. (Fig. 6). Bilateral, focal, or patchy opacities and crazy-paving pattern may be observed in pulmonary hemorrhage. Other than parenchymal opacities, in pulmonary vasculitis, nodules with or without cavitation, centrilobular nodules, a CT halo sign due to perilesional hemorrhage, airway involvement, and pleural effusion may be observed [17, 33]. The reverse halo sign is also reported in granulomatosis with polyangiitis [15]. Associated CT findings, patient history, clinical findings, and serologic tests are essential for differential diagnosis.

### Neoplasms

**Fig. 7:**
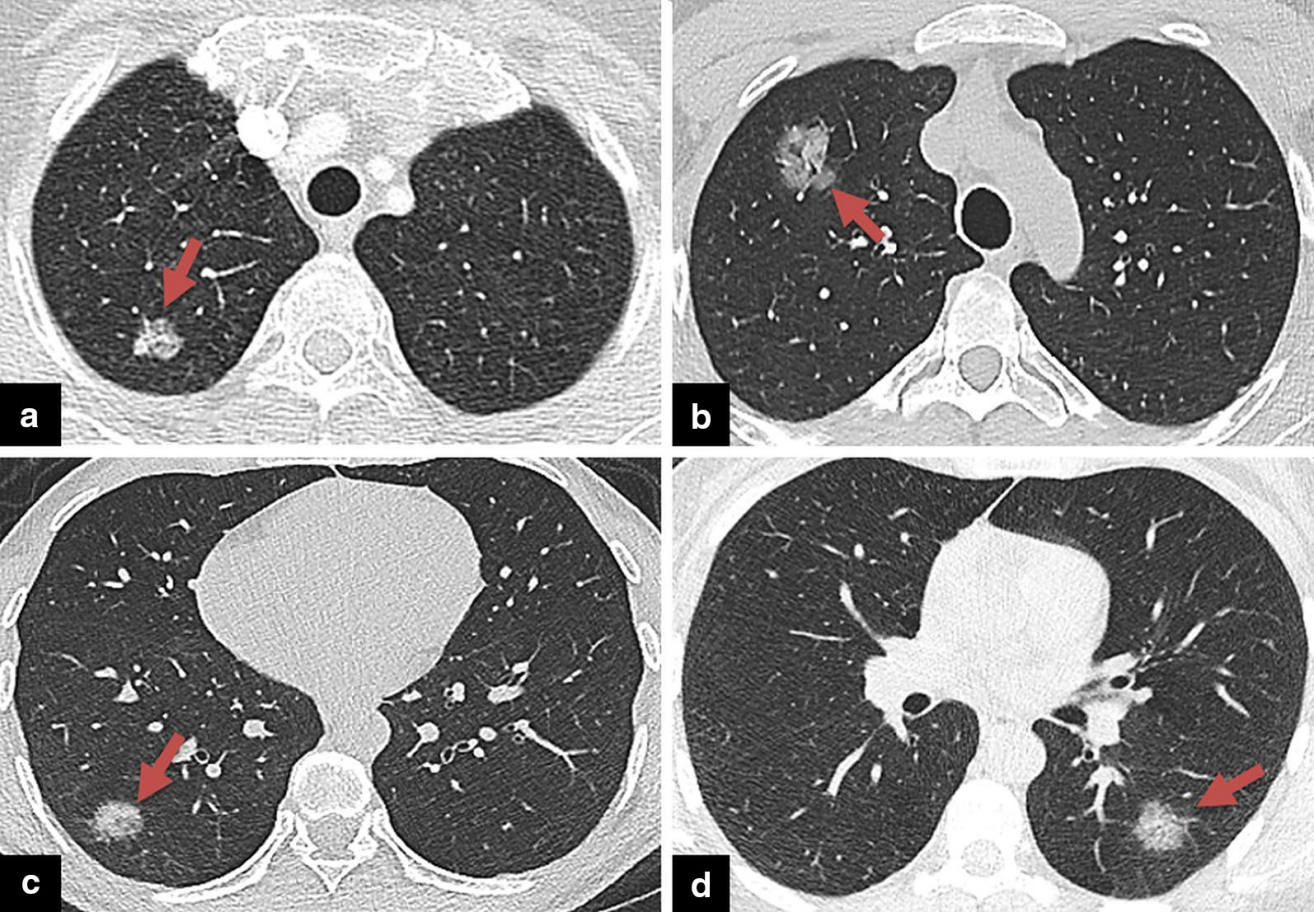
COVID-19 pneumonia-mimicking adenocarcinoma of the lung. **a** Axial chest CT image shows a rounded focal opacity histopathologically proven to be adenocarcinoma in the right upper lobe (arrow). **b**–**d** CT images of three different COVID-19 patients demonstrating unifocal round opacities mimicking adenocarcinoma (arrows)

**Fig. 8:**
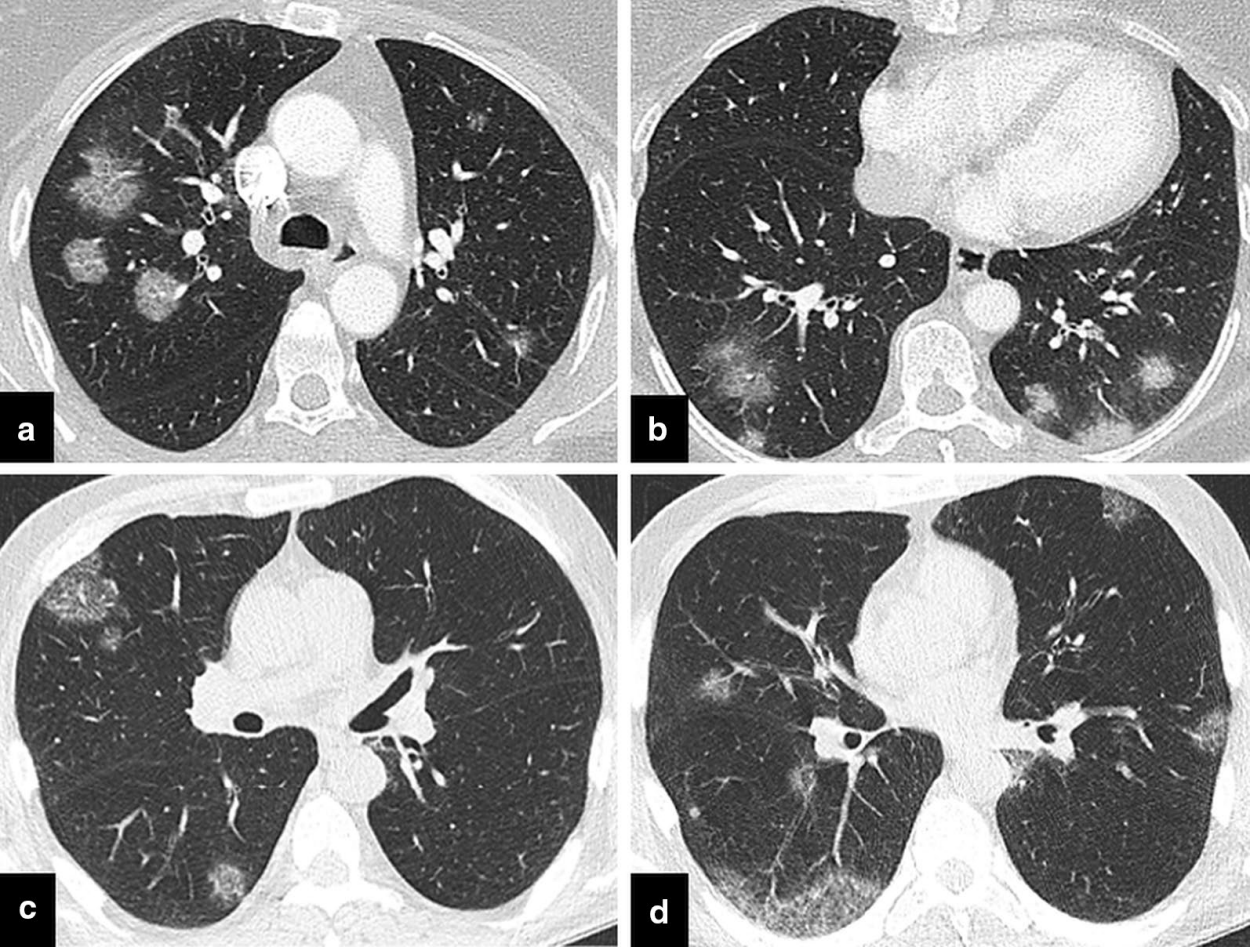
COVID-19 pneumonia-mimicking multifocal adenocarcinoma of the lung. **a**, **b** Multiple randomly distributed GGOs with superimposed septal thickening in both lungs, histopathologically proven to be adenocarcinoma. **c**, **d** Multiple GGOs in a COVID-19 patient with rounded morphology and superimposed septal thickenings mimicking multifocal adenocarcinoma

**Fig. 9:**
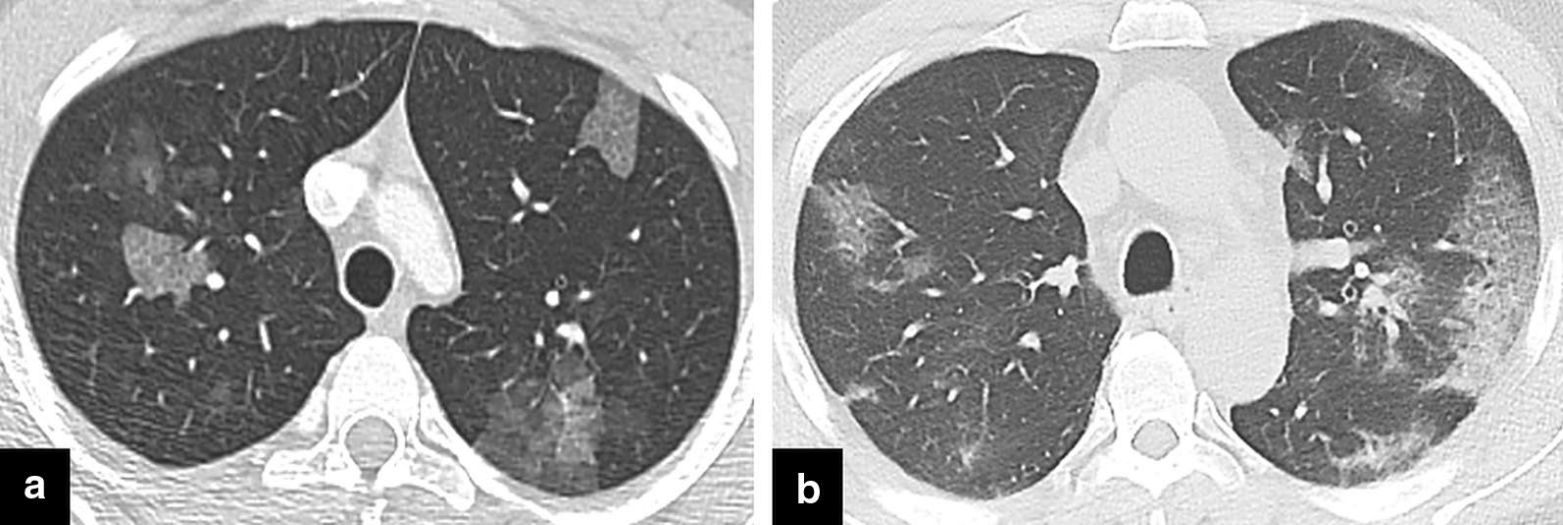
COVID-19 pneumonia mimicking hemorrhagic metastases. **a** Multiple bilateral GGOs with superimposed intralobular septal thickenings in a patient with hemorrhagic epithelioid angiosarcoma metastases. **b** In a patient diagnosed with COVID-19 pneumonia, bilateral multiple GGOs with superimposed intralobular septal thickenings are observed in the upper lobes

Focal GGOs or opacities with rounded morphology may be encountered in COVID-19 pneumonia [10]. In the presence of focal GGOs, neoplastic processes should also be considered. Preinvasive lesions (atypical adenomatous hyperplasia and adenocarcinoma in situ), early-stage adenocarcinoma (Fig. 7), and multifocal adenocarcinoma (Fig. 8) may have variable imaging features, such as pure GGOs, GGOs in combination with consolidation, nodules surrounded by a halo of GGO (CT halo sign), and GGOs with crazy-paving pattern. Air bronchograms in larger lesions, pleural effusion, and lymphadenopathy can be seen [34–37]. Similarly, mucinous adenocarcinoma metastases can manifest as focal or multifocal GGOs, consolidations, and nodules with a CT halo sign [38, 39]. Besides tumoral growth, CT halo sign can represent peritumoral hemorrhage in hemorrhagic metastases such as angiosarcoma [17] (Fig. 9).

### Organizing pneumonia

**Fig. 10:**
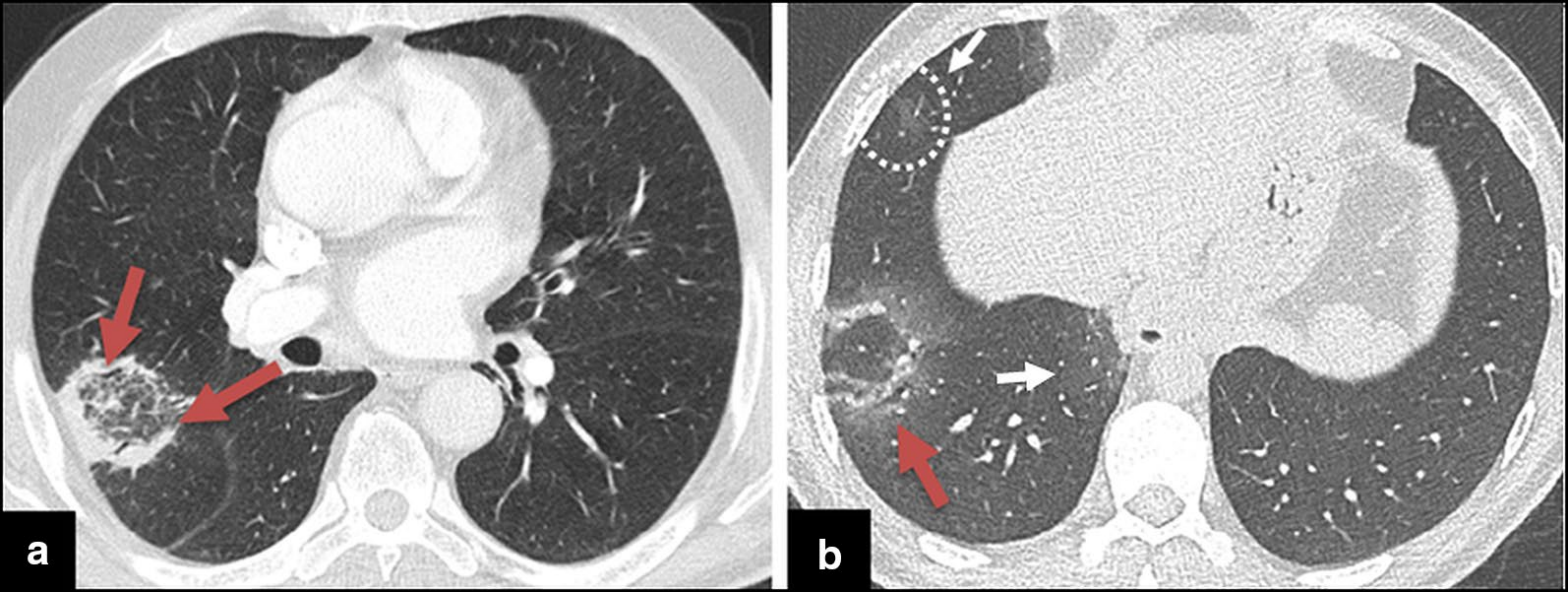
The reverse halo sign in cryptogenic organizing pneumonia and COVID-19 pneumonia. **a** Axial CT image showing organizing pneumonia presenting with reverse halo sign (arrows). **b** A rounded opacity with reverse halo sign and surrounding GGOs in the right lower lobe in COVID-19 pneumonia (arrow). GGOs are seen in the right middle lobe and medial right lower lobe (white arrows)

Organizing pneumonia can be either cryptogenic or secondary to other lung or systemic diseases (infection, drug toxicity, radiotherapy related, vasculitis, collagen vascular diseases, and interstitial lung disease) [40–42]. Typical CT findings are bilateral, asymmetrical, peribronchovascular, and subpleural GGOs and consolidations [41, 43]. Uncommonly, crazy-paving pattern may be seen [36]. Another finding, the reverse halo sign, which was also reported in COVID-19 pneumonia, was firstly described in organizing pneumonia [44] (Fig. 10).

Organizing pneumonia may develop during the course of radiotherapy-induced lung disease (RILD) [45]. GGOs and/or consolidation, with superimposed septal thickening causing a crazy-paving pattern, and a CT halo sign may occur in the early phase of RILD [46, 47].

**Fig. 11:**
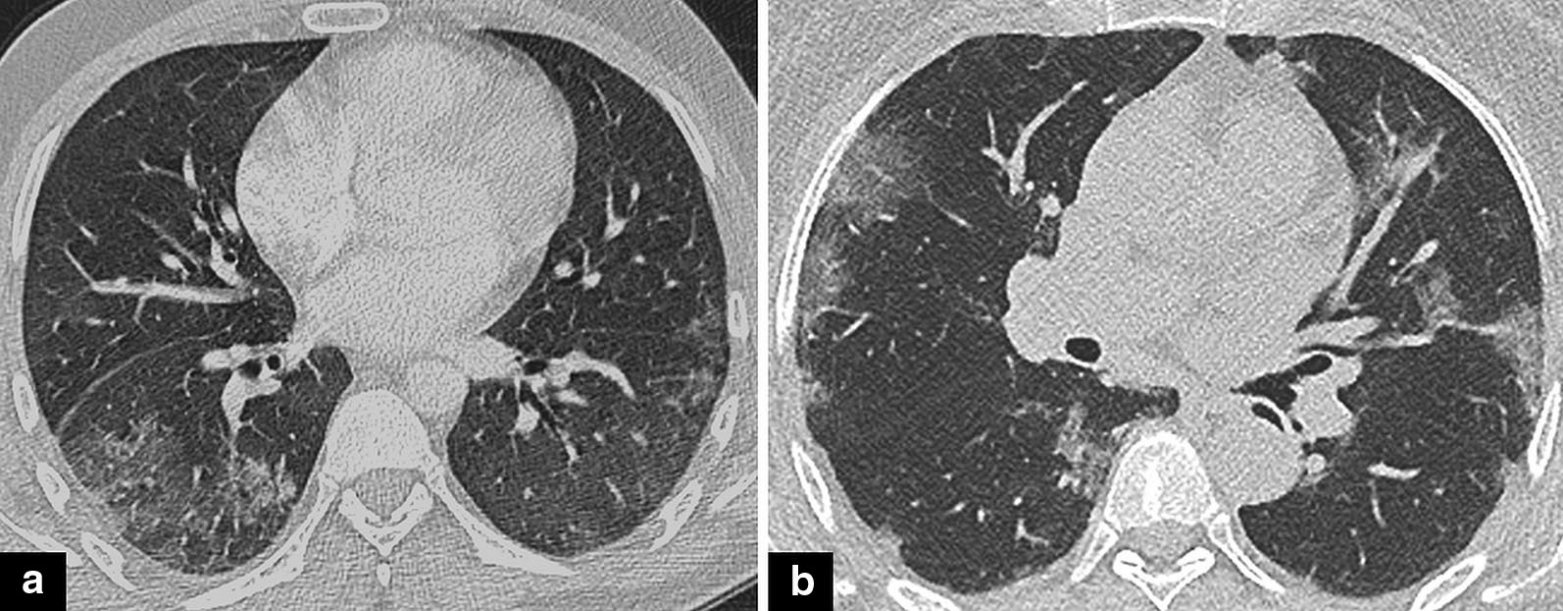
Drug toxicity and COVID-19 pneumonia. **a** Bilateral peripheral GGOs are observed in the axial CT image of a patient receiving bleomycin chemotherapy for testicular malignancy. Following the discontinuation of the drug, opacities were completely resolved. **b** In a patient diagnosed with COVID-19 pneumonia, bilateral multiple GGOs resembling (**a**) are seen

Another cause of secondary organizing pneumonia is drug toxicity. Drug-induced lung diseases may present with variable imaging findings according to the underlying mechanism (hemorrhage, diffuse alveolar damage, edema, and interstitial disease) [48] (Fig. 11).

### Pulmonary alveolar proteinosis

**Fig. 12:**
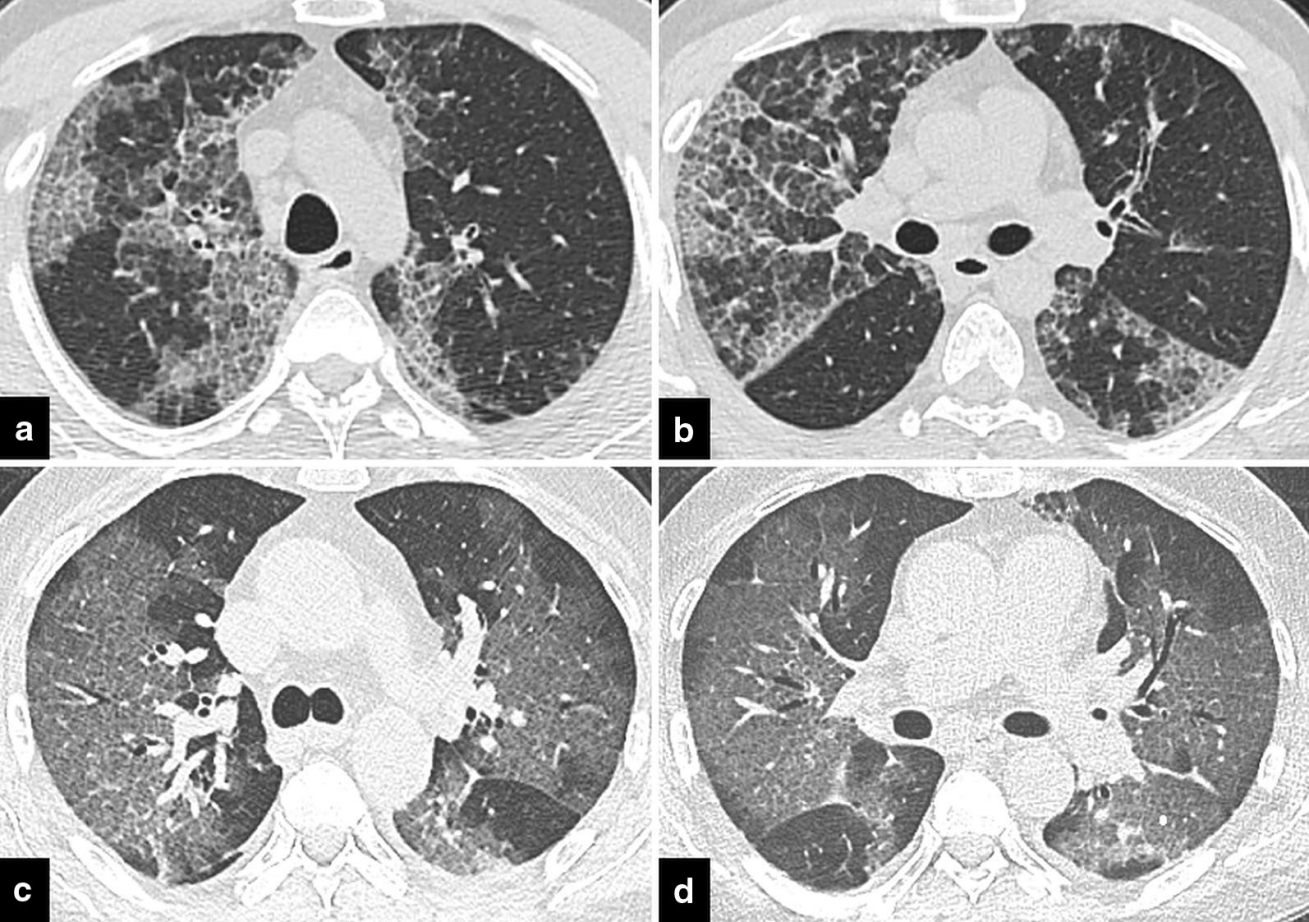
Crazy-paving pattern. **a**, **b** Crazy-paving pattern in pulmonary alveolar proteinosis (PAP). **c**, **d** Axial CT images of a patient with COVID-19 show widespread GGOs with crazy-paving pattern in both lungs

The most common CT findings of pulmonary alveolar proteinosis are bilateral, diffuse, or patchy GGOs and consolidations [49]. The crazy-paving pattern (GGOs superimposed with interlobular and intralobular septal thickening), which was recently reported in COVID-19 pneumonia, was initially described in pulmonary alveolar proteinosis [50] (Fig. 12). Increasing opacities and the presence of the pattern in COVID-19 pneumonia is thought to reflect disease progression [12].

### Sarcoidosis

**Fig. 13:**
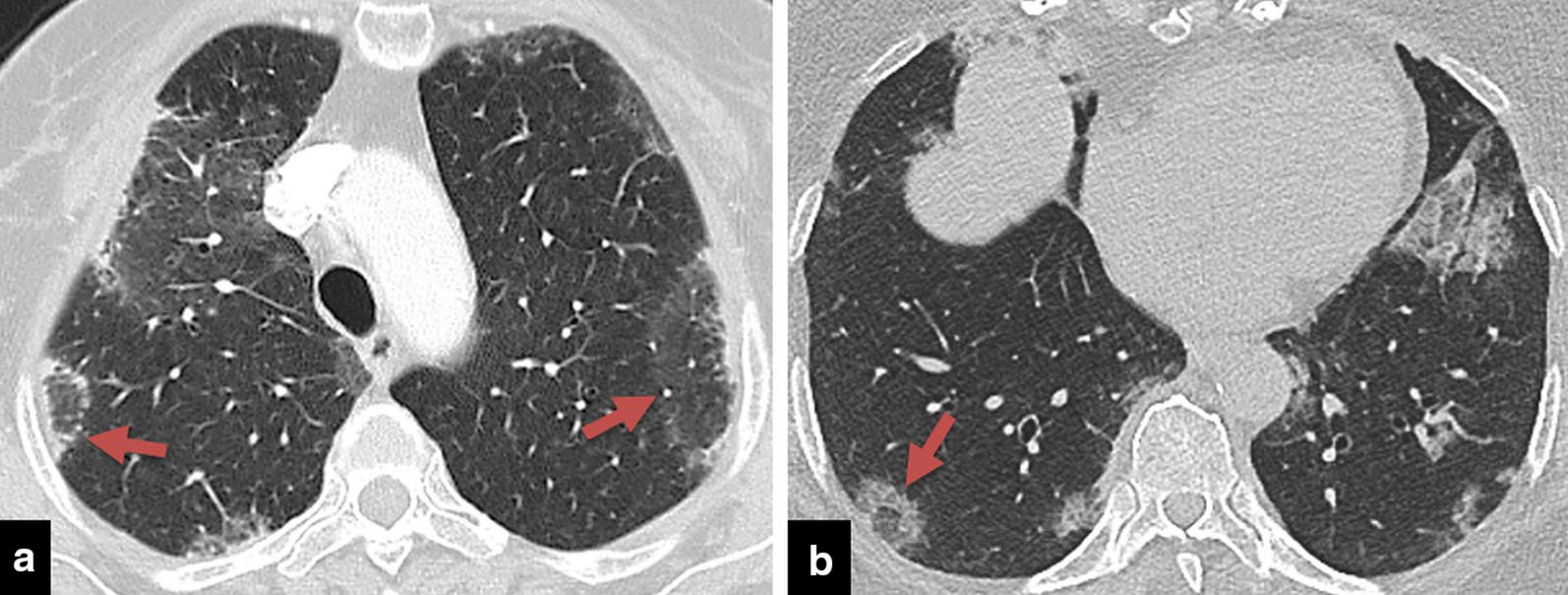
The reverse halo sign in sarcoidosis and COVID-19 pneumonia. **a** Sarcoidosis presenting with organizing pneumonia pattern, peripheral GGOs, and reverse halo signs (arrows). **b** Opacities in COVID-19 pneumonia with reverse halo sign in right lower lobe (arrow). Concomitant multifocal opacities are observed

COVID-19 pneumonia may resemble atypical sarcoidosis in some cases with patchy or nodular/mass-like GGOs, consolidations, a crazy-paving pattern, and a reverse halo sign (Fig. 13). Typical findings of sarcoidosis such as mediastinal and/or bilateral hilar lymphadenopathies and perilymphatic nodular opacities with upper lobe predilection do not match those of COVID-19 pneumonia [51, 52]. In sarcoidosis, the consolidation ring of the reverse halo sign may have nodularities due to the granulomatous process [15].

### Pulmonary infarction

**Fig. 14:**
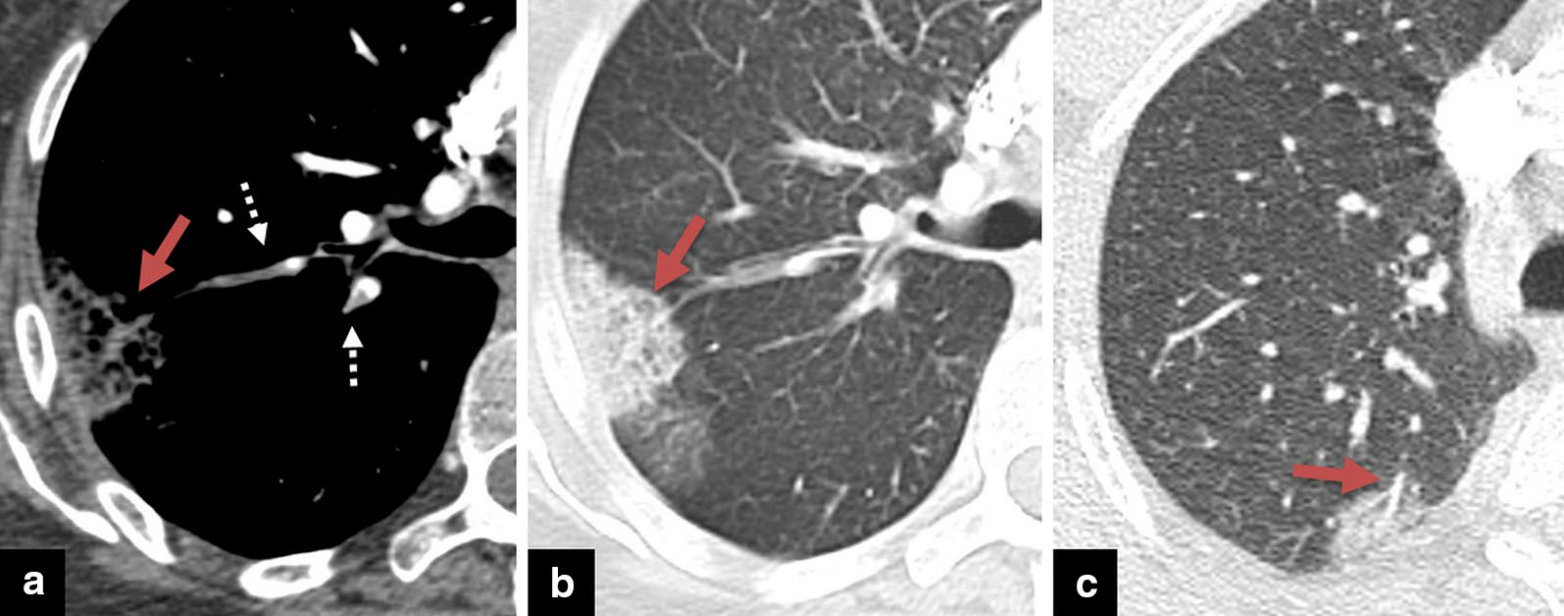
Pulmonary infarction and COVID-19 pneumonia. **a** Axial CTA image in the mediastinal window shows thrombi in pulmonary artery branches (white arrows). **b** In the same patient, pulmonary infarction is seen as a subpleural wedge-shaped opacity in the right lung (arrow). **c** Pulmonary CT angiography was obtained in a patient presented with shortness of breath to the emergency department. CT angiography showed subpleural opacities, but there was no thrombus in the related pulmonary artery branch (arrow). The patient’s COVID-19 RT-PCR result turned out to be positive

Peripheral GGOs and the reverse halo sign of COVID-19 pneumonia may mimic pulmonary embolism-associated parenchymal changes. Preceding peripheral GGOs may also be present in patients with pulmonary embolism before consolidation develops [53]. Supporting CT findings of the pulmonary thromboembolic disease are peripheral wedge-shaped opacities, a reverse halo sign, atelectasis, and the direct visualization of intraluminal filling defects (Fig. 14). Pleural effusion may be seen [15, 54].

An increased incidence of pulmonary thromboembolism is reported recently in COVID-19 pneumonia [55]. Since unenhanced chest CT is used in the routine practice of COVID-19 diagnosis, one should be alert for parenchymal pulmonary infarction findings. If the patient’s condition suddenly deteriorates, pulmonary thromboembolism should be considered, and pulmonary CT angiography may be obtained.

### Interstitial lung diseases

**Fig. 15:**
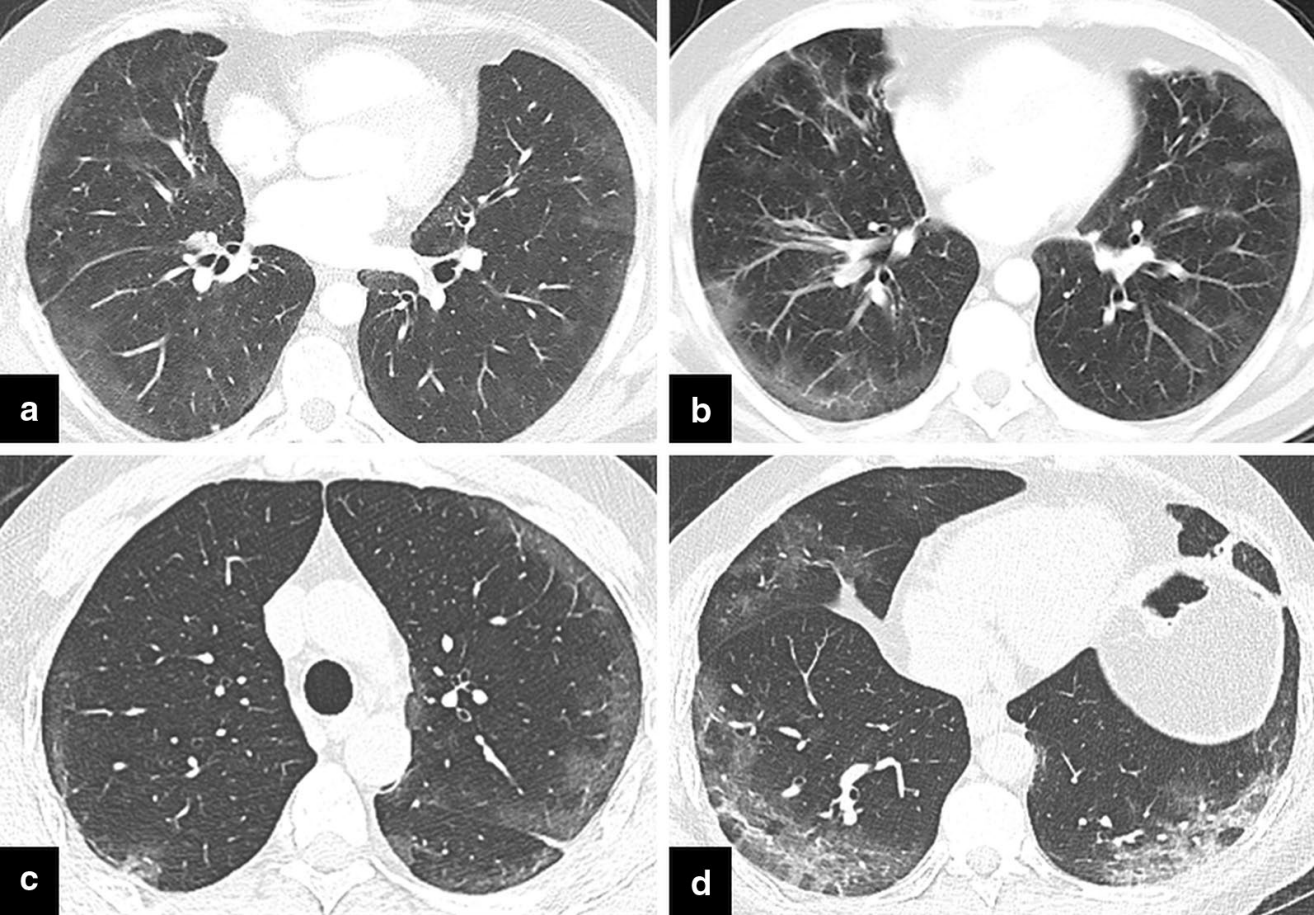
COVID-19 pneumonia-mimicking interstitial lung disease. **a**, **b** In desquamative interstitial pneumonia (DIP), bilateral peripheral GGOs are observed. **c**, **d** Axial CT image showing bilateral subpleural GGOs and opacities in a patient diagnosed with COVID-19

Interstitial lung diseases may also have overlapping CT findings, especially with subacute phase findings of COVID-19 pneumonia. Lower zone and peripherally distributed GGOs are reported in nonspecific interstitial pneumonia (NSIP) and desquamative interstitial pneumonia (DIP) [56, 57]. In NSIP, the crazy-paving pattern and lack of honeycombing might resemble COVID-19 pneumonia [36, 56]. While cystic changes and irregular linear opacities are observed in DIP, honeycombing is also uncommon [57] (Fig. 15).

GGOs are the most common CT finding of lymphocytic interstitial pneumonia (LIP). Unlike in COVID-19 pneumonia, diffuse distribution rather than peripheral distribution, the presence of centrilobular nodules, lymphadenopathy, and cystic airspaces are found in LIP [58].

**Fig. 16:**
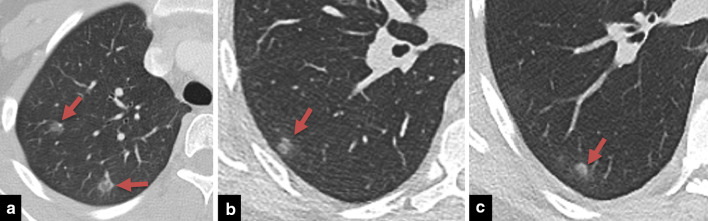
Eosinophilic pneumonia and COVID-19 pneumonia. **a** Nodular GGOs are seen in the right upper lobe in a patient diagnosed with eosinophilic pneumonia (arrows). **b**, **c** Axial CT images of a patient with COVID-19 show nodular GGOs in the right lower lobe (arrows)

Acute eosinophilic pneumonia is characterized by bilateral GGOs and consolidations with random or peripheral distribution, interlobular septal thickening, and a crazy-paving pattern (Fig. 16). Pleural effusion is another commonly reported finding [59].

GGOs with lower zone predominance are also common in subacute hypersensitivity pneumonia. Additional findings such as centrilobular nodules, mosaic perfusion, and air-trapping in expiratory images can help in the differential diagnosis [60].

### Aspiration pneumonia

**Fig. 17:**
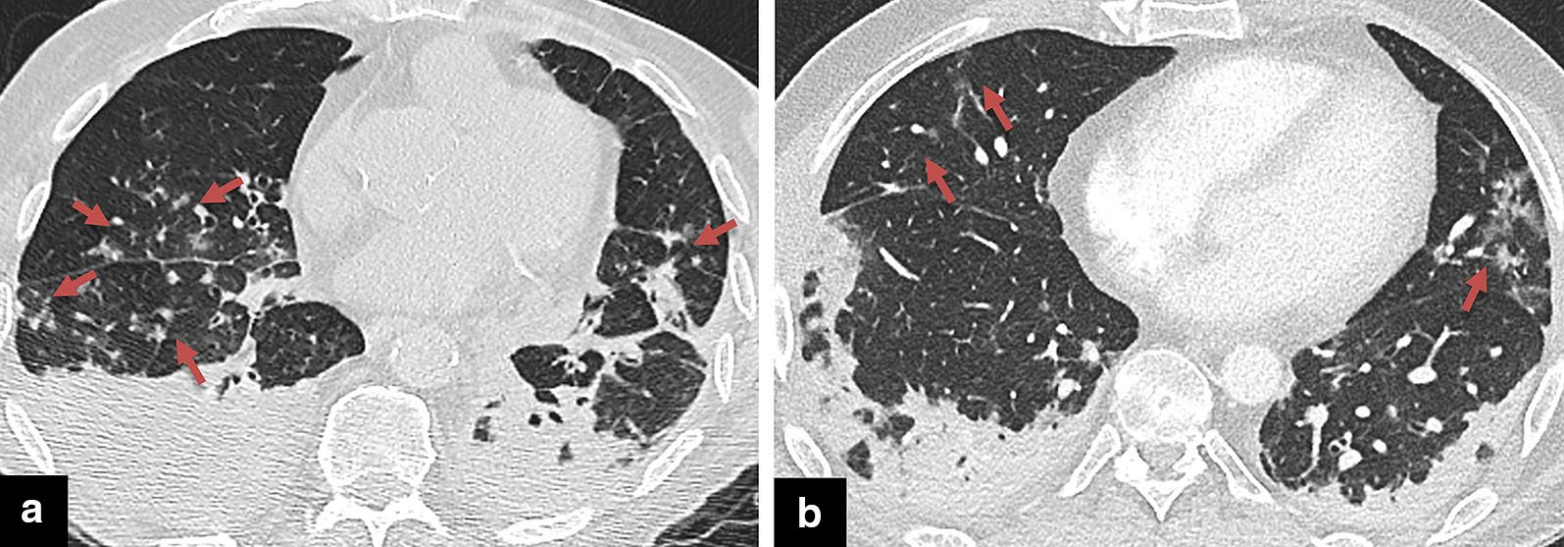
Aspiration pneumonia and COVID-19 pneumonia. **a** The patient had a stroke and was intubated. Axial CT image shows bilateral lower lobe consolidations and centrilobular nodular opacities (arrows) suggestive of aspiration pneumonia. **b** Contrast-enhanced CTA was obtained for suspected pulmonary embolism in a patient diagnosed with COVID-19 pneumonia. CT image shows bilateral consolidations in lower lobes and scattered foci of GGOs (arrows)

Aspiration pneumonia also mostly involves lower lobes and the posterior lung and can manifest as patchy GGOs and/or consolidations. While the findings of bronchiolitis, such as centrilobular nodular opacities and a tree-in-bud pattern, are common in aspiration pneumonia, they are not typically found in COVID-19 pneumonia [61, 62] (Fig. 17). The knowledge of the patient’s pre-existing conditions, general and mental status may help in the differential diagnosis.

## Conclusion

Chest CT is an important tool in the diagnosis of COVID-19 pneumonia with high sensitivity rates. However, CT findings of COVID-19 pneumonia are rather nonspecific and variable during the disease course that may resemble numerous infectious and non-infectious diseases. The awareness and knowledge of the radiological features of these entities are essential in early diagnosis and management of precautions during the pandemic. Duration of the symptoms, background and clinical findings of the patient, ancillary imaging findings, and follow-up CT imaging when needed is helpful in the differential diagnosis.

## Data Availability

Data sharing is not applicable to this article as no datasets were generated or analyzed during the current study.

## Abbreviations

CMV: Cytomegalovirus
COVID-19: Coronavirus disease 2019
CT: Computed tomography
DIP: Desquamative interstitial pneumonia
GGO: Ground-glass opacity
HMPV: Human metapneumovirus
ICU: Intensive care unit
LIP: Lymphocytic interstitial pneumonia
MERS: Middle East respiratory syndrome virus
NSIP: Non-specific interstitial pneumonia
PAP: Pulmonary alveolar proteinosis
PJP: Pneumocystis jirovecii pneumonia
RILD: Radiotherapy-induced lung disease
RSV: Respiratory syncytial virus
RT-PCR: Real-time reverse transcription-polymerase chain reaction
SARS: Severe acute respiratory syndrome virus
SARS-CoV-2: Severe acute respiratory syndrome coronavirus 2
WHO: World Health Organization
